# The complete mitochondrial genome of marine gastropod *Melo melo* (neogastropoda: volutoidea)

**DOI:** 10.1080/23802359.2019.1693293

**Published:** 2019-11-20

**Authors:** Shengping Zhong, Guoqiang Huang, Yonghong Liu, Lianghua Huang

**Affiliations:** a Institute of marine drugs, Guangxi University of Chinese Medicine, Nanning, China;; b Key Laboratory of Marine Biotechnology, Guangxi Institute of Oceanology, Beihai, China

**Keywords:** Mitochondrial genome, *Melo melo*, Nneogastropoda

## Abstract

*Melo melo* is an ecologically and economically important species of Neogastropoda, which is an ecologically diverse group of carnivorous marine gastropods. However, the taxonomic classification and phylogenetic studies have so far been limited. In this study, we report the second complete mitochondrial genome of Volutidae from *M. melo*. The mitogenome has 15,721 base pairs (68.3% A + T content) and made up of total of 37 genes (13 protein-coding, 22 transfer RNAs and 2 ribosomal RNAs), and a control region. This study was the second available complete mitogenomes of Volutidae and will provide useful genetic information for future phylogenetic and taxonomic classification of Neogastropoda.

The order Neogastropoda is a highly diversified group of predatory marine gastropods, which comprise more than 16,000 described species and has adapted to almost every marine environment including the abyssal zone of the oceans (Zou et al. 2011). Neogastropoda contains the most geographically widespread and ecologically diverse families including Muricidae (Zhong et al. 2019a), Nassariidae, Buccinidae (Zhong et al. 2019b) and Volutidae. Many species of Volutidae including *Melo melo* are economically important as luxury seafood and valuable ingredients of traditional medicines (Kanagasabapathy et al. 2011). However, the taxonomy and phylogeny of the Volutidae have been confused due to morphological taxonomic characters convergence and plasticity impacted by environment (Zou et al. 2011). The complete mitochondrial genome is an excellent molecular marker for studying phylogenetic relationships and taxonomy identification, but adequate mitogenome information about the Volutidae is still limited. Here, we report the second complete mitochondrial genome sequence of Volutidae, which will provide a better insight into phylogenetic assessment and taxonomic classification.

A tissue samples of *M. melo* from five individuals were collected from GuangXi province, China (Beihai, 21.44866 N, 109.479636 E), and the whole body specimen (#GR0039) were deposited at Marine biological Herbarium, Guangxi Institute of Oceanology, Beihai, China. The total genomic DNA was extracted from the muscle of the specimens using an SQ Tissue DNA Kit (OMEGA, Guangzhou, China) following the manufacturer’s protocol. DNA libraries (350 bp insert) were constructed with the TruSeq NanoTM kit (Illumina, San Diego, CA) and were sequenced (2 × 150 bp paired-end) using HiSeq platform at BGI Company, China. Mitogenome assembly was performed by MITObim (Hahn et al. 2013). The complete mitogenome of *Cymbium olla* (GenBank accession number: NC_013245) was chosen as the initial reference sequence for MITObim assembly. Gene annotation was performed by MITOS (Bernt et al. 2013).

The complete mitogenome of *M. melo* was 15,721 bp in length (GenBank accession number: MN462590), and containing the typical set of 13 protein-coding, 22 tRNA and 2 rRNA genes, and a putative control region. The overall base composition of the mitogenome was estimated to be A 29.4%, T 38.9%, C 14.3% and G 17.3%, with a high A + T content of 68.3%, which is similar, but slightly higher than *Hemifusus tuba* (68.2%) (Zhong et al. 2019b). The mitogenomic phylogenetic analyses showed that *M. melo* was clustered with *C. olla* within family Volutidae clade with high bootstrap value then clustered with families Nassariidae and Buccinidae (Figure 1), which is consistent with the phylogenetic analyses of Neogastropoda using entire Mitogenome (Cunha et al. 2009). The complete mitochondrial genome sequence of *M. melo* was the second sequenced mitogenome in Volutidae, which will contribute to further phylogenetic and comparative mitogenome studies of Neogastropoda.

**Figure 1.**
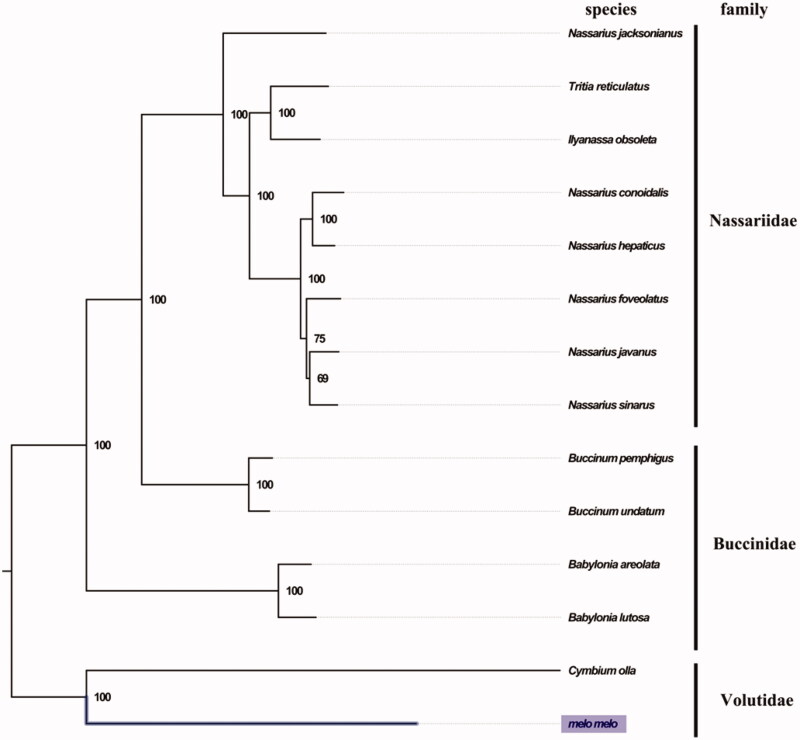
Phylogenetic tree of 14 species in order Neogastropoda. The complete mitogenomes is downloaded from GenBank and the phylogenic tree is constructed by maximum-likelihood method with 100 bootstrap replicates. The bootstrap values were labeled at each branch nodes. The gene's accession number for tree construction is listed as follows: *Babylonia areolata* (NC_023080), *Babylonia lutosa* (NC_028628), *Tritia reticulatus* (NC_013248), *Ilyanassa obsoleta* (NC_007781), *Nassarius conoidalis* (NC_041310), *Nassarius hepaticus* (NC_038169), *Nassarius foveolatus* (NC_041546), *Nassarius javanus* (NC_041547), *Nassarius sinarus* (NC_041545), *Buccinum pemphigus* (NC_029373), *Buccinum undatum* (NC_040940), *Nassarius jacksonianus* (NC_041548), and *Cymbium olla* (NC_013245).
